# Layered Double Hydroxides (LDH) as Delivery Vehicles of a Chimeric Protein Carrying Epitopes from the Porcine Reproductive and Respiratory Syndrome Virus

**DOI:** 10.3390/pharmaceutics16070841

**Published:** 2024-06-21

**Authors:** María José Alonso-Cerda, Mariano J. García-Soto, Arleth Miranda-López, René Segura-Velázquez, José Ivan Sánchez-Betancourt, Omar González-Ortega, Sergio Rosales-Mendoza

**Affiliations:** 1 Facultad de Ciencias Químicas, Universidad Autónoma de San Luis Potosí, Av. Manuel Nava 6, San Luis Potosí 78210, Mexico; a206367@alumnos.uaslp.mx (M.J.A.-C.); mariano.soto@uaslp.mx (M.J.G.-S.); a317919@alumnos.uaslp.mx (A.M.-L.); 2 Sección de Biotecnología, Centro de Investigación en Ciencias de la Salud y Biomedicina, Universidad Autónoma de San Luis Potosí, Av. Sierra Leona 550, San Luis Potosí 78210, Mexico; 3 Unidad de Investigación, Facultad de Medicina Veterinaria y Zootecnia, Universidad Nacional Autónoma de México, Mexico City 04510, Mexico; realselab@gmail.com; 4 Departamento de Medicina y Zootecnia de Cerdos, Facultad de Medicina Veterinaria y Zootecnia, Universidad Nacional Autónoma de México, Mexico City 04510, Mexico

**Keywords:** nanoparticle, nano-vaccine, adjuvant, humoral response, multi-epitopic vaccine

## Abstract

The Porcine Reproductive and Respiratory Syndrome Virus (PRRSV) causes reproductive failure and respiratory symptoms, leading to huge economic losses for the pig farming industry. Although several vaccines against PRRSV are available in the market; they show an overall low efficacy, and several countries have the need for vaccines covering the local, circulating variants. This project aims at developing a new chimeric antigen targeting specific epitopes from PRRSV and evaluating two test adjuvants to formulate a vaccine candidate. The test antigen was called LTB–PRRSV, which was produced recombinantly in *Escherichia coli* and consisted of the heat labile enterotoxin B subunit from *E. coli* (LTB) and four epitopes from PRRSV. LTB–PRRSV was rescued as inclusion bodies and methods for its solubilization, IMAC-based purification, and refolding were standardized, leading to mean yields of 18 mg of pure protein per liter culture. Layered double hydroxides (LDH) have been used as vaccine adjuvants given their biocompatibility, low cost, and positive surface charge that allows an efficient adsorption of negatively charged biomolecules. Therefore, LDH were selected as delivery vehicles of LTB–PRRSV. Pure LTB–PRRSV was adsorbed onto LDH by incubation at different LDH:LTB–PRRSV mass ratios (1:0.25, 1:0.5, 1:1, and 1:2) and at pH 9.5. The best adsorption occurred with a 1:2 mass ratio, and in a sucrose-tween solution. The conjugates obtained had a polydispersity index of 0.26, a hydrodynamic diameter of 192 nm, and a final antigen concentration of 64.2 μg/mL. An immunogenicity assessment was performed by injecting mice with LDH:LTB–PRRSV, Alum/LTB–PRRSV, or LTB–PRRSV in a scheme comprising three immunizations at two-week intervals and two dose levels (1 and 5 μg). LTB–PRRSV was capable of inducing strong humoral responses, which lasted for a longer period when LDH was used as the delivery vehicle/adjuvant. The potential of LDH to serve as an attractive carrier for veterinary vaccines is discussed.

## 1. Introduction

The porcine reproductive and respiratory syndrome (PRRS) is caused by a virus with high genetic variability. This disease is prevalent worldwide and it is estimated that 60–80% of the swine population is positive to the causative agent [1], the porcine reproductive and respiratory syndrome virus (PRRSV), generating prominent economic losses around the world estimated at 560 millions of US dollars per year [2]. Therefore, PRRS prevention strategies are highly relevant, since the virus has a high transmission rate, and it is difficult to identify infected or asymptomatic pigs [1]. There are two PRRSV strains: European and North American, which show a 60% genomic similarity. Mexico is the country with the greater number of reports on PRRSV outbreaks with >80% of the cases in worldwide databases in 2020 [3]. PRRSV is a small virus (50–72 nm) conformed by a small capsid (20–30 nm) [1]. At the genomic level, it has seven open reading frames (ORF) encoding for five proteins, from which the GP5 and M proteins play a key role in pathogenesis. The N protein has antigenic regions and possesses a conserved region between the two strains [4]. GP5 is the subunit with highest genetic variability and one of the most important for vaccine design given its role during infection, mediating the formation of a heterodimer that binds vimentin and heparan sulfate at the cell surface, with the subsequent recruitment of CD169, CD161, CD209, and CD151 to achieve virus entry [5].

The main transmission routes of PRRSV are the airways, contact with contaminated materials and equipment, and the placental route, and its transmission often occurs in the weaning zone, reproduction zone, and the common areas of pig farms. The infection begins in the nasal and endometrial epithelia, where macrophages are invaded, which allows the virus to reach the blood stream leading to systemic infection and development of signs and symptoms [1]. One prevention strategy is the isolation or elimination of infected animals; however, the most effective approach to fight against PRRSV is vaccination. The PRRSV vaccines currently in the market include formulations based on modified live virus (MLV), inactivated virus (KIV), DNA, viral vectors, and recombinant proteins, which show an overall acceptable efficacy. Multiple vaccines have been used in the fight against PRRSV, having efficacy to reduce the clinical disease. Another limitation comes with adjuvants, since these are often protected by patents, and this represents a restriction for the manufacturing of new vaccines. Therefore, developing proprietary adjuvanted vaccines is a priority for the field to ensure autonomous production. Moreover, the current PRRSV vaccines achieve protection primarily against homogeneous circulating strains and, since PRRSV is a virus with high genetic variability, the commercial vaccines are often ineffective for local outbreaks, especially when vaccines produced in other countries are used. Therefore, the main challenges for the current PRRSV vaccines comprise reaching complete immuno-protection and achieving long-lasting immunity, especially in sows [6,7].

Despite this, the genetic variability of the pathogen can generate situations where the vaccines do not cover the circulating strains in particular areas. For this reason, there is a need to develop vaccines covering circulating strains in particular regions, and one approach to achieve this goal is multi-epitope vaccine design, in which conserved protective epitopes from a key antigen could lead to high protection against the circulating strains [8].

This approach also allows simplification of the production process, since no pathogen is handled, offering lower costs and enhanced safety for personnel. Moreover, side effects overall are lower when subunit vaccines are applied. In fact, attenuated vaccines against PRRSV have been associated with adverse effects such as immuno-dysregulation [9,10].

However, the main challenge for developing subunit vaccines comes with immunogenicity, which is often lower when compared to the whole pathogen given their low complexity. Therefore, adjuvants acting as delivery systems and immunostimulatory compounds should be contemplated to achieve a proper immunogenic formulation. The major role of the adjuvants is to enhance the immune response evoked by the vaccine antigen in terms of magnitude and breath [11]. The main categories of adjuvants available in the market include emulsions, polymeric materials, saponins, and cytokines [12]. Layered double hydroxides (LDHs) are synthetic, anionic clays with general formula [M^II^_1−x_M^III^_x_(OH)_2_]^x+^[A^n−^_x/n_/]×yH_2_O, where M^II^ indicates divalent metal cations, such as Mg^2+^, Ca^2+^, Zn^2+^, Cu^2+^, Co^2+^, or Ni^2+^, and M^III^ denotes trivalent metal cations, such as Fe^3+^, Al^3+^, Ga^3+^, or Cr^3+^. Hydrated organic or inorganic A^n−^ ions exist in the interlamellar space (such as CO_3_^2−^, NO_3_^−^, SO_4_^2−^, OH^−^, Cl^−^, or Br^−^) [13]. LDH can be obtained by several procedures, which include coprecipitation, sol–gel methods, hydrothermal synthesis, urea hydrolysis, ion exchange, calcination–rehydration, self-assembly, hydrothermal/solvothermal, in situ chemical reduction, and mechanochemical procedures [14]. In recent years, a two-step synthesis (coprecipitation–hydrothermal) has been optimized, resulting in LDH nanoparticles with size between 60 and 150 nm, with good yields and crystallinity [15]. LDH have been explored for several biomedical applications. For instance, LDH have been reported as excellent delivery vehicles for several drugs [16,17,18], scaffolds in tissue regeneration (particularly in bone), and materials of dental use [19,20]. Moreover, LDH have also been suggested as vaccine adjuvants. In 2018, Chen et al. demonstrated the capacity of LDH to form nodules that facilitate cellular uptake, antigen release, and active recruitment by the immune cells. Li et al. 2022 reported a major immune response, with LDH as adjuvant when compared to aluminum hydroxide. Despite this, LDH produced a slighter inflammatory response at the injection site [21,22]. Our group has recently reported a systematic synthesis and characterization protocol for LDH and confirmed that LDH acted as adjuvant for BSA as model antigen [19]. Herein, a new chimeric protein targeting PRRSV was produced in recombinant *E. coli* and used to formulate a vaccine candidate in which LDH was applied as putative adjuvant. The proposed formulation was evaluated in test mice to determine its immunogenicity in terms of the magnitude of the humoral response induced, providing relevant data for subunit vaccine development against PRRSV.

## 2. Materials and Methods

### 2.1. Gene Design

Epitope selection was based on an analysis of 183 sequences for the PRRSV ORF5 gene accessed at the Genbank, which correspond to field isolates obtained in Mexico, along with relevant strains including the reference strain VR2332. Highly conserved epitopes were identified using Mega 7 and the ConSurf Server. Transmembrane domains were determined by the following software: SMART 3.4, HMMER 3.0, and TMHMM 2.0, while signal peptide identified using Lipo P1.0, SignalP 4.1, and SMART SIGNAL 3L. Once the signal peptide sequences were eliminated, and using only sequences that were not located trans-membranely, the identification of B epitopes was carried out using the following software: ABC PRED, available at http://crdd.osdd.net/raghava/abcpred/, accessed on 17 June 2024; Bcpred, available at http://ailab.cs.iastate.edu/bcpreds/, accessed on 17 June 2024; and Antigenic EpiQuest Suite 4.1. Candidate sequences were analyzed with DNASTAR Protean to determine their secondary structure and hydrophobicity, while the Peptide Property Calculator tool was used to determine the physical–chemical characteristics of the epitopes.

A chimeric protein called LTB–PRRSV was designed to display the four selected epitopes from GP5. The chimeric arrangement comprises the B subunit from the heat labile *E. coli* enterotoxin (LTB) followed by a GPGP linker and the target epitopes in tandem. A synthetic gene encoding the LTB–PRRSV protein was designed based on the codon usage for *E. coli* by Biomatik Inc. (Kitchener, ON, Canada) and cloned into the pET-15b vector, which drives the transgene expression under the T7 promoter controlled by the lac operator. These plasmids have an ampicillin-resistant gene.

### 2.2. LTB–PRRSV Antigen Production

Chemically competent *Rosetta* DE3 *E. coli* cells were transformed with the pET15b-LTB–PRRSV vector and plated on Agar–Luria Broth (LB) supplemented with ampicillin (100 mg/mL) and chloramphenicol (40 mg/mL), incubating overnight at 37 °C. Three colonies showing resistance to antibiotics were selected and propagated in selective LB medium to assess protein expression. An inoculum was generated for each of the colonies selected by inoculating them in 10 mL of selective LB medium, followed by incubation at 37 °C for 24 h at 180 rpm. The inoculums were grown at 37 °C until the OD_600_ was between 0.6 and 0.8. An aliquot of the culture was withdrawn before protein induction as a negative control. The number 2 colony was selected, and protein expression was subsequently induced by adding lactose followed by incubation at 28 °C for 16 h at 180 rpm. The biomass was recovered by centrifugation at 4 °C for 10 min at 7000 rpm.

### 2.3. Protein Extraction and SDS–PAGE Analysis

The biomass recovered from the expression assays was frozen at –80 °C, and the cells were subsequently lysed by sonication using a sonicator at 70% amplitude with 9 on/off cycles of 30 s. The lysate was centrifuged at 7000 rpm for 20 min, and the supernatant (soluble fraction) was separated from the pellet (insoluble fraction). Soluble and insoluble protein fractions were analyzed by SDS–PAGE using 16% acrylamide gels. Each lane was loaded with 30 µL of the corresponding sample previously mixed with 6 µL of 5× loading buffer and boiled for 10 min at 95 °C to denature proteins. The analysis was run at 160 V for 80 min and the gel was subsequently stained with Coomassie solutions [23].

### 2.4. Solubilization and Purification of LTB–PRRSV

The insoluble fraction obtained from 1 L culture was subjected to several washing steps followed by centrifugation at 7000 rpm and 10 min at 4 °C. The pellet was first washed three times with 5 mL of deionized water and subsequently washed twice with 5 mL of PBS plus 1% Triton X-100 (subjected to sonication at 70% amplitude with 2 pulses of 30 s and 20 min of rest); finally, a PBS washing was applied. For solubilization of the inclusion bodies, the pellet was treated with a buffer containing 20 mM Na_2_HPO_4_ at pH 7.4 with 4 M urea, and centrifugation was applied to collect the supernatant. The remnant pellet was treated with a buffer containing 20 mM Na_2_HPO_4,_ 500 mM NaCl, 8 M urea at pH 7.4 (buffer A), the mixture rested for 30 min and a new centrifugation step was applied. The remnant pellet was treated overnight with the latter buffer and after centrifugation the supernatant was recovered. The solubilized proteins recovered after each step were stored at –20 °C until further analysis by SDS–PAGE. The last supernatant was used for protein purification using IMAC.

A 2 mL IMAC column (with immobilized Ni^2+^ ions) equilibrated with buffer A was used. A 1 mL sample was injected at a flow rate of 0.25 mL/min. Despite not being adsorbed, an important amount of recombinant protein was recovered free of impurities in the flow-through fraction.

### 2.5. Refolding and Concentration of LTB–PRRSV

The LTB–PRRSV antigen was subjected to a refolding process using buffers with decreasing urea concentration (from 8 to 0 M). The sequence of buffers is shown in Table 1.

The refolded protein was analyzed by SDS–PAGE and quantified by densitometry (Image J 1.53) with BSA as standard. The purified antigen was concentrated using an Amicon Ultra-15 Centrifugal Filter (10 kDa MWCO).

### 2.6. Synthesis of LDH Nanoparticles (LDH NP)

The synthesis of LDH consisted in the coprecipitation of magnesium nitrate and aluminum nitrate with sodium hydroxide, followed by the hydrothermal treatment of the produced suspension [15]. Into 15 mL of 0.4 M NaOH under vigorous vortex mixing, 10 mL with 0.15 M Mg(NO_3_)_2_∙6H_2_O and 0.05 M Al(NO_3_)_3_∙9H_2_O were quickly poured, keeping the coprecipitate under mixing for 10 min. This suspension was washed twice by replacing the supernatant from centrifugation (10 min, 21,200× *g*) with deionized water and resuspending the pellet with an ultrasonic tip (5 s, 20% amplitude). The washed suspension was placed in a PTFE-lined stainless-steel reactor for its hydrothermal treatment in an oven (2 h, 100 °C), allowing it to reach room temperature afterward. The treated suspension was washed once as described earlier to obtain a definite LDH NP suspension.

The concentration of LDH was estimated after centrifuging aliquots of 1 mL, discarding the supernatant, and drying the pellets in a desiccator (24 h, 25 °C). The particle size (d_z_) and ζ potential were measured with a Zetasizer Pro particle analyzer (Malvern Panalytical, Malvern, UK). The morphology was characterized with a JEM-2100 electron microscope (JEOL Ltd., Akishima, Japan).

### 2.7. Bioconjugation of LTB–PRRSV with LDH NP

The conjugation of LDH with LTB–PRRSV involved preconditioning both in Tris buffer at pH 9.5 to favor the adsorption of the antigen on monodisperse NP, while testing different mass ratios to formulate stable conjugates with the required amount of antigen for each dose. The antigen LTB–PRRSV in a solution of 10% sucrose with 0.01% Tween 20 at pH 6 was dialyzed against 20 mM Tris buffer at pH 9.5 using a cellulose membrane (3.5 kDa MWCO. The LDH NP, originally in water as a 4 mg/mL suspension, were diluted and adjusted to be in 20 mM Tris buffer at pH 9.5, readily dispersed for their conjugation with the dialyzed antigen.

LDH NP (0.2 mg/mL) and LTB–PRRSV (400, 200, and 100 µg/mL), both in 20 mM Tris at pH 9.5, were combined in equal volumes to render LDH/antigen mass ratios of 1:2, 1:1, and 1:0.5, which were continuously mixed in a rotary agitator (1 h, 30 rpm). Each conjugate was washed by centrifuging (10 min, 21,200× *g*) and dispersing the pellet in water, while the supernatant was collected to quantify non-bound antigen. Each washed conjugate was finalized by centrifuging again and dispersing the pellet in a solution of 10% sucrose with 0.01% Tween 20 at pH 6, while discarding the supernatant.

The antigen adsorbed on LDH (C_s_) and the non-bound remaining in the supernatant (C_e_) were compared with antigen at the same initial concentration (C_0_) as the conjugates. The first two were quantified by UV–vis and all three with SDS–PAGE. The particle size (d_z_) of the finalized conjugate was measured with a Zetasizer Pro (Malvern).

### 2.8. Immunization

BALB/c mice (10–12-week-old) were used to evaluate the immunogenicity of LTB–PRRSV under approval of the Institutional Ethics Committee (CEID-2015-048). Mice were randomly divided into 3 groups (*n* = 4) and subjected to a subcutaneous (s.c.) immunization scheme comprising three doses (200 µL) on days 0, 14, and 28. The groups received one of the following treatments: (1) 5 µg of LTB–PRRSV in 10% sucrose plus 0.01% Tween 20, (2) 5 µg of LTB–PRRSV:LDH conjugate, (3) 5 µg of LTB–PRRSV + Al(OH)_3_, or (4) the vehicle alone. Al(OH)_3_ (G Biosciences, St. Louis, MO, USA, cat no. 786-1215) was used at a ratio of 1:5. Blood samples were withdrawn on days 27, 42, 56, and 86 to measure seric levels of antibodies.

### 2.9. Antibody Levels by Enzyme Linked Immunosorbent Assay (ELISA)

The levels of IgG were determined by indirect ELISA using the LTB–PRRSV protein as target antigen and performing three washes with PBS 1× between each incubation step. ELISA plates were coated with 200 ng of the antigen/well by overnight incubation at 4 °C using carbonate buffer (15 mM Na_2_CO_3_ + 35 mM NaHCO_3_ at pH 9.6). Wells were blocked for 2 h at 25 °C with a 5% fat-free milk solution prepared in PBS. Serial dilutions of the test sera were added by triplicate and plates incubated overnight at 4 °C. The plates were subsequently incubated for 2 h at room temperature with a peroxidase-conjugated anti-mouse IgG secondary antibody (1:2000). Finally, antibody binding was detected using an ABTS substrate solution (0.6 mM 2,20-azino-bis (3-ethylbenzothiazoline-6-sulfonic acid) + 0.1 M citric acid + 1 mM H_2_O_2_ at pH 4.35). After 60 min of incubation at 25 °C, optical density values were measured at 405 nm using a MultiskanR FC microplate photometer (Thermo Fisher Scientific, Waltham, MA, USA).

### 2.10. Statistical Analysis

The data obtained for antibody measurements were analyzed via one-way analysis of variance using the GraphPad Prism^®^ software version 10. Statistical significance was set at *p* < 0.05.

## 3. Results

### 3.1. Production of LTB–PRRSV

**Figure 1:**
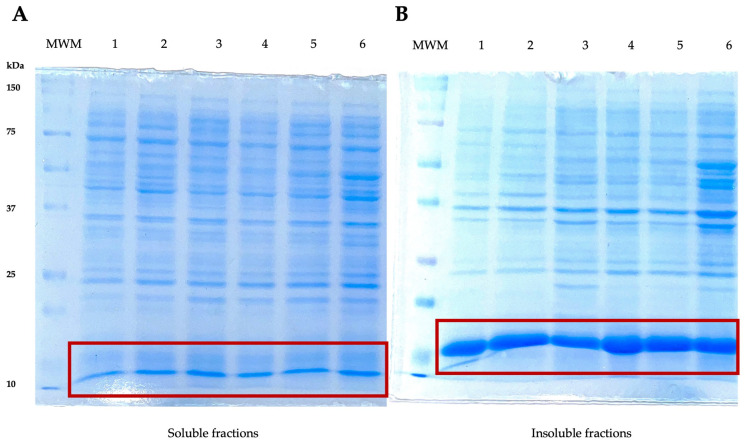
SDS–PAGE analyses of soluble and insoluble protein fractions during the production of LTB–PRRSV (16 kDa). (**A**) Soluble fractions. MWM, molecular weight marker; 1, soluble fraction from colony 1; 2, soluble fraction from colony 2; 3, soluble fraction from colony 3; 4, 5, and 6, duplicates of soluble fractions from colonies 1, 2, and 3, respectively. (**B**) Insoluble fractions. MWM, molecular weight marker; 1 insoluble fraction from colony 1; 2, insoluble fraction from colony 2; 3, insoluble fraction from colony 3; 4, 5, and 6, duplicates of insoluble fractions from colonies 1, 2, and 3, respectively.

The expression of the recombinant LTB–PRRSV protein was performed in *E. coli* cultures having OD_600_ of 0.6–0.8 under the following conditions: 15 g/L of lactose, 16 h of expression period, and 28 °C at 180 rpm. The recombinant antigen LTB–PRRSV has a theoretical molecular weight of 16 kDa and it was produced as inclusion bodies (Figure 1). Despite assessing different expression conditions (e.g., lower temperature and lactose concentration), the expression of LTB–PRRSV as soluble protein was not observed and protocols for the solubilization and refolding of the target protein were established.

### 3.2. LTB–PRRSV Solubilization

**Figure 2:**
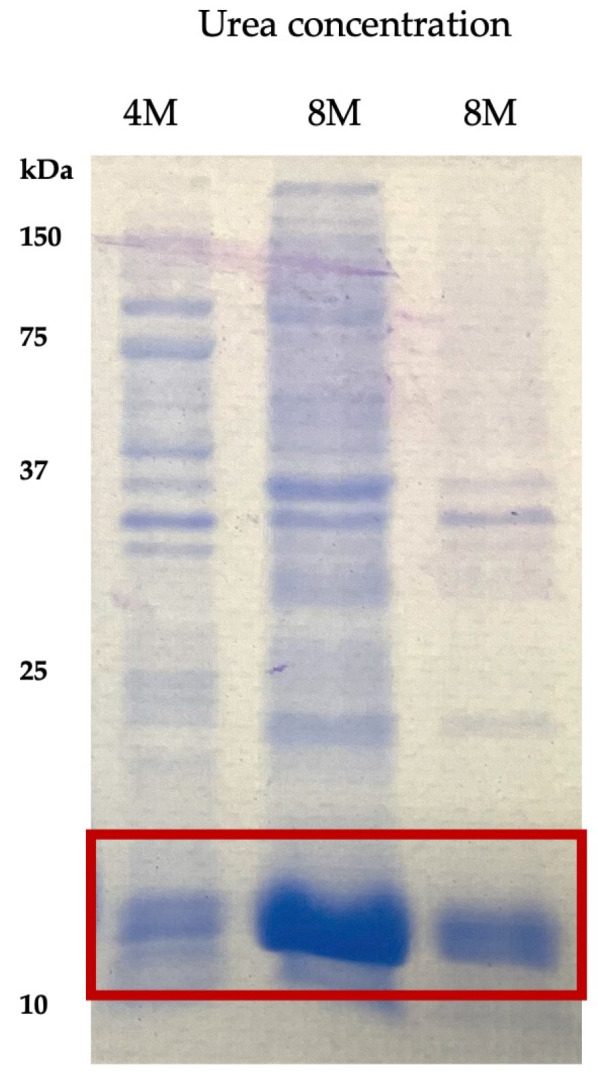
Extracts of LTB–PRRSV solubilized with urea solutions at different concentrations. Lane 1, LTB–PRRSV solubilized during 30 min with a 4 M urea solution; Lane 2, LTB–PRRSV solubilized during 30 min with an 8 M urea solution; Lane 3, LTB–PRRSV solubilized overnight with an 8 M urea solution.

LTB–PRRSV was solubilized using different buffers with varying urea concentration and the extracts containing urea at 4 and 8 M were analyzed by SDS–PAGE (Figure 2). The buffer containing 8 M urea led to a high rate of solubilization after a 30 min treatment, with the presence of several contaminant proteins. In contrast, the overnight treatment led to a fraction with lower contamination and, therefore, this fraction was selected for further purification. The purity level of LTB–PRRSV in this raw solubilized protein was 85%.

### 3.3. LTB–PRRSV Purification

**Figure 3:**
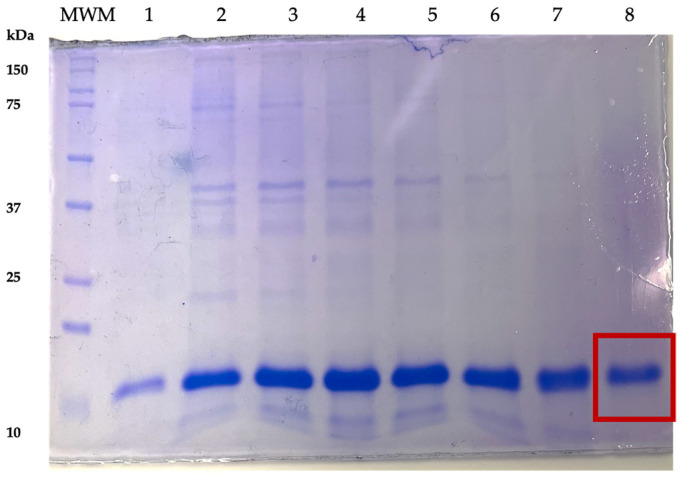
Fractions from the LTB–PRRSV IMAC-based purification. Lane MWM, molecular weight marker; Lanes 1–8, unbound fractions collected upon column washing. Fraction 8 was used for the refolding step, having a 92% purity.

The purification of LTB–PRRSV was accomplished using IMAC. Figure 3 shows the flow-through fractions (collected every 2 min), the impurities leave the column earlier, which allows obtaining a highly pure protein in the late fractions. The purity of the last fraction was 92% and this fraction was used for refolding.

### 3.4. LTB–PRRSV Refolding

The refolding of LTB–PRRSV was initially tested with buffers reported in the literature and solutions used in our research group [24]; however, LTB–PRRSV precipitated under these approaches. The sequence of buffers reported in Materials and Methods was the combination that was successful since no precipitation occurred in any of the buffers. 1 mM 2-mercaptoethanol was included to provide a reducing environment. After refolding, LTB–PRRSV was analyzed by SDS–PAGE in which a standard curve prepared with BSA (50–500 µg/mL) was included, allowing the quantification of the protein of interest, revealing that the LTB–PRRSV concentration was 400 μg/mL, which represents a yield of 18 mg of pure refolded protein per liter of fermentation culture.

### 3.5. LDH NP Synthesis and Bioconjugation with LTB–PRRSV

**Figure 4:**
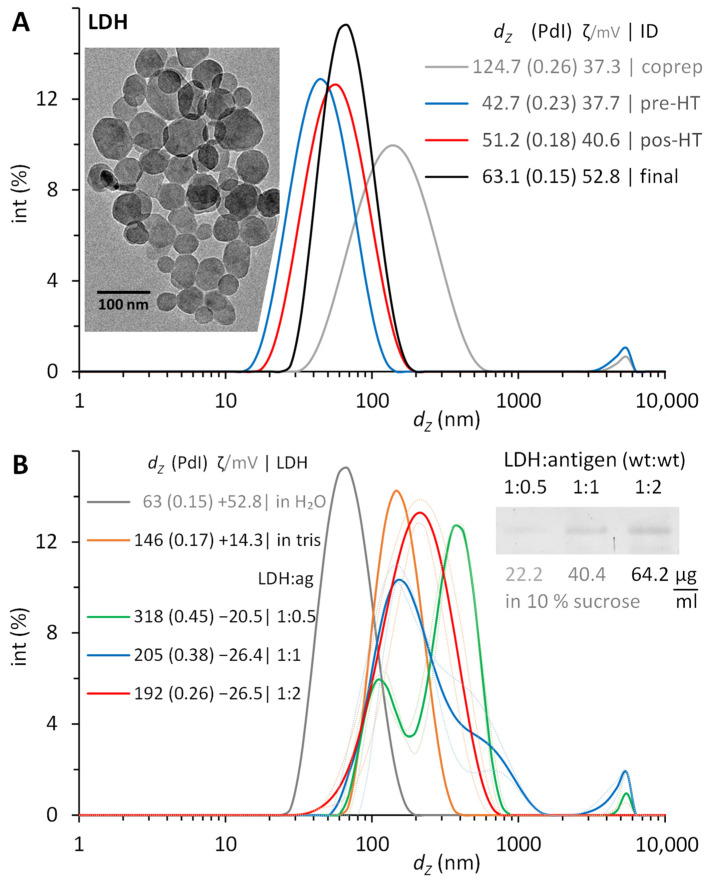
Layered double hydroxide (LDH) NP synthesized and then conjugated with recombinant LTB–PRRSV. (**A**) Size distributions during consecutive stages of refinement after their synthesis by coprecipitating a 3:1 molar ratio of Mg^2+^:Al^3+^. Washing twice prior (pre-HT) their hydrothermal treatment (HT) and washing once after this (post-HT) results in monodispersed LDH NP. Insert: TEM image of a typical final suspension. (**B**) Size evolution of LTB–PRRSV/LDH after the physisorption of increasing amounts of antigen protein with mass ratios from 1:0.5 to 1:2 during 1 h at room temperature. Insert: PAGE image of antigen adsorbed on LDH. Hydrodynamic diameter (d_Z_), polydispersity index (PdI), and ζ potential of LDH synthesized and suspended in water, LDH suspended in 20 mM Tris (pH 9.5), and of LDH conjugated with antigen (LDH/ag mass ratios of 1:0.5, 1:1, 1:2) and suspended in 10% sucrose containing 0.01% Tween 20.

The synthesis with a 3:1 molar relation of Mg^2+^:Al^3+^ resulted in white turbid suspensions of LDH, which were refined by washing via centrifugation and ultrasonic resuspension. After washing the coprecipitate once, the NP measured 125 nm, with a polydispersity index (PdI) of 0.26. A second wash produced a more compacted pellet with NP of 43 nm and PdI still above 0.2. Narrower size distributions were secured after their hydrothermal treatment, with PdI of 0.18, and further to 0.15 in the final suspension after a third wash (Figure 4A).

In all syntheses, the size remained around 50 nm after twice washing the coprecipitate and minimally increased by 10 nm after the thermal treatment and a final washing. Similarly, the ζ potential reached +40 to +50 mV (indicating good stability), while the PdI decreased below 0.2 (indicating monodispersed NP). The TEM images showed hexagonal platelets with diameters within the particle size distribution measured by dynamic light scattering (DLS). The LDH NP here synthesized were reproducible and comparable with that reported earlier [19], matching those (63.1 nm, 0.15 PdI) produced after 2 h of thermal treatment.

The ζ potential of the LDH in water (pH 6) is positive (+52.8 mV), which implies the presence of a surface with positive charge. Moreover, the point of zero charge (PZC) for LDH has been reported previously as 11 [25]. The theoretical isoelectric point of LTB–PRRSV is 8.5. Therefore, we decided to conduct the adsorption experiments at a pH of 9.5 to favor the electrostatic interaction between the antigen and the nanoparticles. At this pH of 9.5 with 20 mM Tris, the protein would be negatively charged, while the LDH NP had a positive ζ potential (+14.3 mV). Once completed, the LDH/LTB–PRRSV conjugates had a negative ζ potential, which had a higher value with larger concentrations of antigen adsorbed.

We also tested pH values of 9.0 and 10.5, but the adsorption was significantly reduced in the former and non-existent in the latter. For the case of the pH set to 10.5, a carbonate buffer was used, and it was noticed that the ζ potential of the LDH changes to a negative value due to carbonate adsorption, which not only hinders protein adsorption but promotes aggregation of the LDH NP. After conducting the adsorption of LTB–PRRSV onto LDH under different mass ratios, there were lower concentrations of antigen produced conjugates with higher average hydrodynamic diameter and increased PdI. The use of a 1:2 ratio provided the higher adsorption rate, leading to a particle size of 192 nm with a PdI value of 0.26, and an antigen concentration adsorbed onto the particles of 64.2 μg/mL (Figure 4B). Considering the initial size and concentration of LDH for the conjugation, and LTB–PRRSV covering an estimated area of 8.3 nm^2^, the concentration of this antigen to form a full monolayer on LDH would be 62 μg/mL. Therefore, the mass relations of 1:0.5, 1:1, 1:2 here tested would provide antigen to form the equivalent to 0.8, 1.6, and 3.2 monolayers. As the adsorption reaches equilibrium, not all the protein is physiosorbed, and typically high concentrations are used to secure enough antigen on the nanoparticles to stabilize it. The 1:2 ratio here employed was sufficient to fully coat the LDH NP with LTB–PRRSV, providing good stability, while having the non-adsorbed antigen at a minimum.

The original LDH are nanoparticles stabilized electrostatically. When adsorbing protein molecules on their surface, this stabilization is perturbed, but the conjugate needs to remain stable. Since the protein molecule is a charged polymer with hydrophobic regions on its surface, it is mandatory for the conjugate to be stable (to prevent agglomeration and/or aggregation), which implies that the combination of steric and electrostatic stabilization surpasses the attractive forces of the hydrophobic areas. For the 1:0.5 ratio, the conjugate increases its size from 146 to 318 nm (both in 10% sucrose with 0.01% Tween 20), while the PdI increases from 0.17 to 0.45. Moreover, three signals appear on the size distribution (Figure 4). This is a clear indication of not enough protein on the surface of the LDH to prevent their interaction. As expected, increasing the proportion of antigen reduces the size and PdI of the conjugate with the best results for the 1:2 ratio, where a single peak in the size distribution is obtained. In the case of the ζ potential, irrespective of the ratio used, a change from a positive to a negative value is observed upon protein adsorption (Figure 4B).

The LTB–PRRSV:LDH conjugates obtained under this condition were subjected to a stability evaluation in four different buffers by measuring particle size to estimate the formation of agglomerates. The solution of 10% sucrose with 0.01% Tween 20 resulted in the higher stability and thus it was used as the test vaccine formulation containing 5 μg of the target antigen.

### 3.6. Immunization Assay

**Figure 5:**
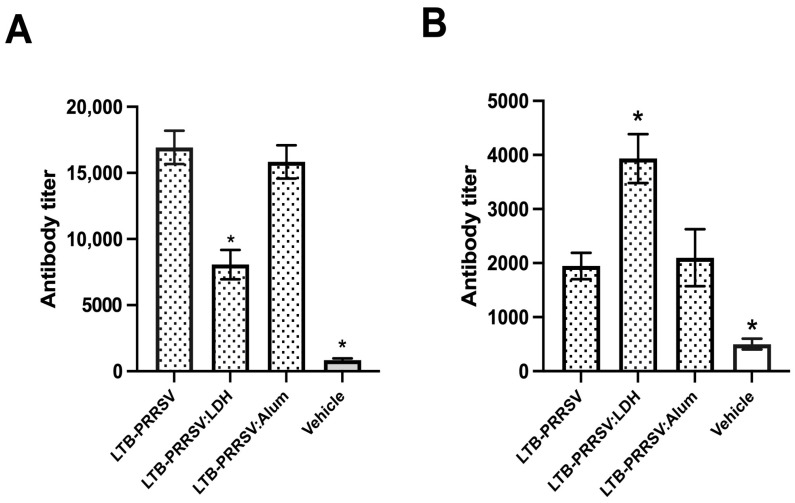
Humoral response induced by the LTB–PRRSV antigen using different formulations. Mice groups (*n* = 4) were s.c. injected on days 0, 14, and 28 with 5 µg of LTB–PRRSV alone (LTB–PRRSV), 5 µg of LTB–PRRSV/LDH conjugate (LTB–PRRSV:LDH), 5 µg of LTB–PRRSV + Al(OH)_3_ (LTB–PRRSV:Alum), or the vehicle alone (Vehicle). Blood samples were withdrawn on days 42 (**A**) and 86 (**B**) to measure seric anti-LTB–PRRSV IgG levels by ELISA. The asterisks denote statistical differences (*p* < 0.05) versus the LTB–PRRSV/Alum group as control.

A mice immunization assay was performed to assess the immunogenicity of the soluble LTB–PRRSV antigen and compare it with the LTB–PRRSV/LDH conjugate and LTB–PRRSV adsorbed onto alum particles. The administration of soluble LTB–PRRSV antigen led to strong IgG responses measured on day 42 (Figure 5A), with the group adjuvanted with alum having a similar response, while the group adjuvanted with LDH showed a lower potency (*p* < 0.05).

Antibody measurements were also performed on day 86 (Figure 5B) to assess the induction of long-lasting responses, revealing that the treatment with LTB–PRRSV/LDH induced a response that prevailed at this timepoint over those showed by the antigen alone or adjuvanted with alum. Therefore, it is proposed that LDH modifies the immunogenic properties of LTB–PRRSV.

## 4. Discussion

This study aimed at proposing an innovative approach to generate vaccines against PRRSV, using a safe carrier capable of enhancing the uptake and processing of the antigen for the induction of robust adaptive immune responses. A chimeric antigen called LTB–PRRSV was produced in *E. coli,* recovering it as insoluble protein. The establishment of solubilization and refolding protocols allowed a system with yields of 18 mg of refolded protein per liter of culture that was amenable for immunization trials in mice. The concentration obtained of LTB–PRRSV post-refolding (430 μg/mL) was similar to other reports for the production of recombinant antigens in *E.coli* [26] and higher than that reported for another microalgal expression system with level expressions around 2 μg/g [27], but lower than *Clostridium botulinum* (205 mg/kg of cells) and *Pichia pastoris* (25 mg/L) [28]. However, the refolding step is laborious and future studies could be focused on approaches to achieve the expression of LTB–PRRSV as a soluble protein, which will decrease production time and cost and improve product homogeneity and scalability [29].

The attenuated or modified virus vaccines currently on the market against PRRSV are effective only for infection by homologous strains; however, they do not provide effective protection when the infection is generated by heterologous strains, which are the most common among pigs [30]. In the literature, there are reports where subunit vaccines have shown an efficient immune response in swine, allowing the stimulation of specific immune responses. This type of vaccine has been shown to increase IFNγ+, CD4+, and CD8+ levels. However, for the protection of this type of vaccine to be complete, activation of the humoral and cellular response is necessary [31].

We performed the conjugation of the target antigen to LDH to evaluate if this carrier enhances the immune response against it. The adsorption capacity of LDH for this target antigen is considered adequate for vaccine formulation, since the amount of antigen usually applied during immunization is in the order of micrograms and this can be easily captured in a reasonable amount of LDH. According to the literature, LDH has shown a good capacity to achieve conjugation with proteins such as BSA. Starting with LDH of 56 and 88 nm, the LDH-BSA conjugates reported had antigen concentrations of 73 and 93 μg/mL for a mass ratio of 1:5, with final sizes of 154 and 244 nm and PdI near to 0.2 [19]. These results are similar to those obtained in this study with an antigen concentration of 64.2 μg/mL in a mass ratio of 1:2 with sizes of 196 nm and PdI of 0.26. Other reports have shown that LDH NP has an adsorption capacity of 0.54 mg of ovalbumin (OVA) per mg LDH NP. In contrast, the adsorption of OVA on LDH nanosheets (LDH NS) is higher, with 2.38 mg of OVA per mg of LDH NS [32]. The maximum reported adsorption capacity of BSA was 0.70 mg per mg of LDH in a study where different conditions were assessed to optimize the stability of the conjugates (pH, mass ratio, LDH particle size, and anions intercalated in the LDH) [33]. For the LTB–PRRSV/LDH conjugate, a value of 0.32 mg per mg LDH NP was obtained.

As expected, the sizes of the conjugates depend on the dispersing medium and the stability provided by the adsorbed antigens. For example, intimin β adsorbed on LDH changed their size from the nanoscale (107–115 nm) to the microscale (>1000 nm) when the conjugates were placed in PBS [34]. Other reports show LDH in nanoscale with sizes ranging from 100 to 300 nm; however, these sizes were measured before the conjugation [35,36]. In this study, the LDH-PRRSV/LDH conjugate aggregated in PBS with the size increasing from 192 nm to >1500 nm, which is not favorable for biological applications, since this size does not favor an efficient uptake by immune system cells.

One of the challenges for the proposed LTB–PRRSV:LDH nano-vaccine will come with scaling-up the production process at low costs since, for veterinary applications, a very low cost per dose is required to make the technology viable in the field [37].

Once the LTB–PRRSV:LDH conjugate was obtained, we proceeded with the immunogenicity assessment. Since in a previous work [19] we have validated that LDH is a safe material (no significant reduction on the viability of HEK293T cells was induced when exposed to LDH at a 6.25–500 µg/mL concentration range), we performed the in vivo evaluation of the conjugate with an LDH concentration of 39 µg/mL, which lies within the range validated as safe.

The immunogenicity data from mice studies revealed that LTB–PRRSV is capable of inducing strong systemic IgG response without adjuvants, which is in agreement with the adjuvant effects extensively reported for epitopes fused to LTB [38,39]. However, the tested adjuvants improved the quality and robustness of the induced immune response in the short term as revealed by the IgG levels measured two weeks after the last boosting. Importantly, when long lasting immune responses were measured (6 weeks after the last boosting), a differential behavior was observed with the LTB–PRRSV:LDH group having a response that sustained higher antibody levels when compared to the formulations adjuvanted with Alum or lacking adjuvant. The fact that the mice treated with LTB–PRRSV:LDH in this work did not present any obvious toxicity sign suggest that the vaccine is safe. Further studies will comprise evaluating tissue damage at the injection site and monitorization of hematologic parameters, as well as weight gain and food and water consumption as detailed safety parameters of the vaccine.

The mechanisms by which LDH modulate immune cells mechanisms have not been clearly elucidated [13,40,41]. It has been reported that LDH forms deposits upon injection that remain for at least 35 days, allowing the generation of an inflammatory response that involves the recruitment and maturation of antigen presenting cells and the release of 60–70% of the cargo, leading to stronger immune responses [42,43]. The capacity of LDH to act as adjuvant was demonstrated using BSA as target antigen in mice, which were treated with LDH-BSA conjugates varying in sizes and dose levels; it was found that the use of LDH and Alum led to stronger humoral responses compared to BSA alone. However, when long-lasting responses were evaluated on day 115, only the use of LDH allowed the achievement of sustained antibody levels, which is an indicator that this adjuvant promotes a more mature immune response, with the LDH-BSA 56 treatment, as that which induced the higher antibody titers, surpassing the response achieved with Alum [19]. Similar findings have been reported for other inorganic nanocarriers, such as mesoporous silicon particles (PSiP), which were co-administered with a peptide derived from the RAGE receptor associated to Alzheimer’s disease, allowing the induction of higher humoral responses when compared to those induced when the Freund´s adjuvant was used; therefore, this nanomaterial stands as a potential adjuvant/carrier for vaccinology [44].

The immune system regulation exerted by nanomaterials is complex and could be considered as a function of particle size, charge, functionalization approach, and the nature of the antigenic molecule. LDH has also been found to promote an anti-inflammatory response associated to the secretion of IL-6 and TNF-α, while reducing platelet aggregation and leukocyte migration [45]. However, detailed knowledge of the mechanisms behind the adjuvant effects of LDH should be generated to expand and optimize their application in vaccinology. Our research group is currently performing transcriptional analysis to contribute to this field. Moreover, safety is a crucial aspect for vaccine development and, although no obvious toxicity effects were detected in the test cell lines and mice, future studies should focus on evaluating local inflammation at the administration site, bioaccumulation, and other safety parameters, such as hematologic parameters, serum biochemistry, antinuclear and neutralizing antibody detection, weight gain, and food intake [46].

The next stage of this study will be focused on determining the protective potential of the immune responses induced by the LTB–PRRSV/LDH conjugates, which will be critical to justify the assessment of this vaccine in a clinical trial to determine the safety and efficacy in pigs.

## 5. Conclusions

LDH have been applied in the development of a nano-vaccine candidate against PRRSV, which was based on the chimeric protein LTB–PRSSV obtained at convenient yields in recombinant E. coli, yielding a stable refolded antigen successfully used for nano-vaccine development based on the adsorption on the LDH NP. The LTB–PRRSV/LDH conjugates stand as a promising nano-vaccine candidate in terms of stability and immunogenicity, since this nano-vaccine showed a superior capacity for the induction of strong, long-lasting humoral responses.

## Figures and Tables

**Table 1:** Buffers composition to refold LTB–PRRSV.

Buffer	Composition
1	6 M Urea, 20 mM Tris-base, 1 mM 2-mercaptoethanol, and 0.01% Tween 20.
2	4 M Urea, 20 mM Tris-base, 1 mM 2-mercaptoethanol, 1 mM cysteine, 0.01% Tween 20, and PBS 1×
3	2 M Urea, 20 mM Tris-base, 1 mM 2-mercaptoethanol, and 0.01% Tween 20.
4	20 mM Tris-base, 1 mM 2-mercaptoethanol, and 0.01% Tween 20.
5	10% sucrose and 0.01% Tween 20.

## Data Availability

Data will be made available upon request.
